# Normocalcaemic hyperparathyroidism and primary hyperparathyroidism: least significant change for adjusted serum calcium

**DOI:** 10.1530/EJE-20-0634

**Published:** 2020-10-27

**Authors:** Marian Schini, Richard Jacques, Eleanor Oakes, Nicola Peel, Jennifer S Walsh, Richard Eastell

**Affiliations:** 1 Department of Oncology and Metabolism, University of Sheffield, Sheffield, UK; 2 School of Health and Related Research (ScHARR), University of Sheffield, Sheffield, UK; 3 Sheffield Teaching Hospitals National Health Service Foundation Trust (STH NHS FT), Sheffield, UK

## Abstract

**Introduction:**

The least significant change (LSC) is a term used in individuals in order to evaluate whether one measurement has changed significantly from the previous one. It is widely used when assessing bone mineral density (BMD) scans. To the best of our knowledge, there no such estimate available in the literature for patients with disorders of calcium metabolism. Our aim was to provide an estimate of the least significant change for albumin-adjusted calcium in patients with normocalcaemic hyperparathyroidism (NPHPT) and primary hyperparathyroidism (PHPT).

**Methods:**

We used the within-subject standard deviatio calculated in a population of NPHPT and PHPT patients and multiplied it by 2.77.

**Results:**

The LSC for NPHPT and PHPT were found to be 0.25 and 0.24 mmol/L, respectively (1.00 and 0.96 mg/dL). In clinical practice, the value of 0.25 mmol/L could be used.

**Discussion:**

The least significant change given, could be used in two ways in these patients. First, it gives a range to which values are expected. This can provide some reassurance for the patient and the physician in cases of intermittent hypercalcaemia. Moreover, it can be a marker of whether an individual has an actual significant change of his calcium after parathyroid surgery.

## Manuscript

Normocalcemic hyperparathyroidism (NPHPT) is a disorder of calcium metabolism which is characterised by persistently normal calcium levels and consistently elevated parathyroid hormone (PTH) values. According to the latest workshop on asymptomatic primary hyperparathyroidism (PHPT), the PTH measurement should be confirmed on at least two consecutive measurements, while normal calcium on several occasions. Moreover, other causes of secondary hyperparathyroidism have to be excluded; these include medications known to affect PTH levels, low vitamin D, chronic kidney disease, renal calcium loss (hypercalciuria) and diseases of the gastrointestinal tract known to affect calcium absorption (1, 2, 3).

NPHPT is usually encountered during the laboratory evaluation of osteoporosis and has been associated with consequences known to affect patients with PHPT (4, 5). There has been an increasing number of studies published recently, which show a potential benefit of parathyroid surgery (6, 7, 8, 9, 10).

A recent study published from our group identified a number of patients with NPHPT in a tertiary centre and showed that patients with NPHPT usually have intermittent hypercalcemia, as do PHPT patients. This study supported the hypothesis that NPHPT is a variant of PHPT (11). We estimated the adjusted serum calcium in these patients using the method of Barth *et al*.** (12) and the variability observed over time complicates their monitoring. According to the Fourth International Workshop on asymptomatic PHPT, calcium and PTH should be tested every year and if there is a progression to hypercalcemia, surgery could be an option (13). However, most of these patients have intermittent hypercalcemia; the subsequent results might be confusing to whether surgery should be recommended.

The least significant change is a term used in everyday practice in order to evaluate whether one measurement has significant change from the previous one. It is widely used when assessing bone mineral density (BMD) scans. The International Society for Clinical Densitometry (ISCD) recommends calculating the LSC for a 95% CI, which is done by multiplying the precision error by 2.77 (https://www.iscd.org). In the case of laboratory evaluations, this would be done by using the within-subject standard deviation (14). To the best of our knowledge, there no such estimate available in the literature for patients with calcium metabolism disorders. Our aim was to provide an estimate of the least significant change in patients with NPHPT and PHPT.

In our previous study, we had a group of 11 NPHPT patients which were defined as having normal albumin-adjusted calcium and high parathyroid hormone (PTH), both on at least two consecutive occasions, normal estimated glomerular rate (eGFR: ≥60 mL/min/1.73 m^2^) and vitamin D replete (≥ 50 nmol/L) on the index day (11). For this population, we used an approach described by Bland & Altman (14) to calculate the within-subject standard deviation and the 95% CIs for adjusted calcium. This was found to be 0.089 (0.080, 1.000) mmol/L (11). In order to perform this analysis, the assumption that standard deviations across the subjects are similar should be made. However, this is not true in all clinical situations. Using a single ‘mean’ standard deviation might underestimate the variability for some patients (15). We understand that this is a limitation of this approach and that the true within-subject standard deviation might be bigger. A suggested approach to overcome this problem is to use the 90th centile of the within-subject variance. This method is considered to be a conservative approach (15). This approach would give a within-subject standard deviation of 0.115 mmol/L. Therefore, the least significant changes from these estimates for patients with NPHPT would be 0.25 and 0.32 mmol/L, respectively.

There was a group of 17 patients with PHPT in our study, defined as high albumin-adjusted calcium and high PTH on at least two consecutive occasions, having normal eGFR and being vitamin D replete on the index day. We excluded patients having familial hypocalciuric hypercalcaemia (FHH). Full details can be found in our study (11). For these patients, the respective within-subject SD was found to be 0.088 (0.079, 0.097) (11), while when using the more conservative approach, this would be 0.112. Hence, the least significant changes from these estimates would be 0.24 and 0.31 mmol/L, respectively.

**Figure 1:**
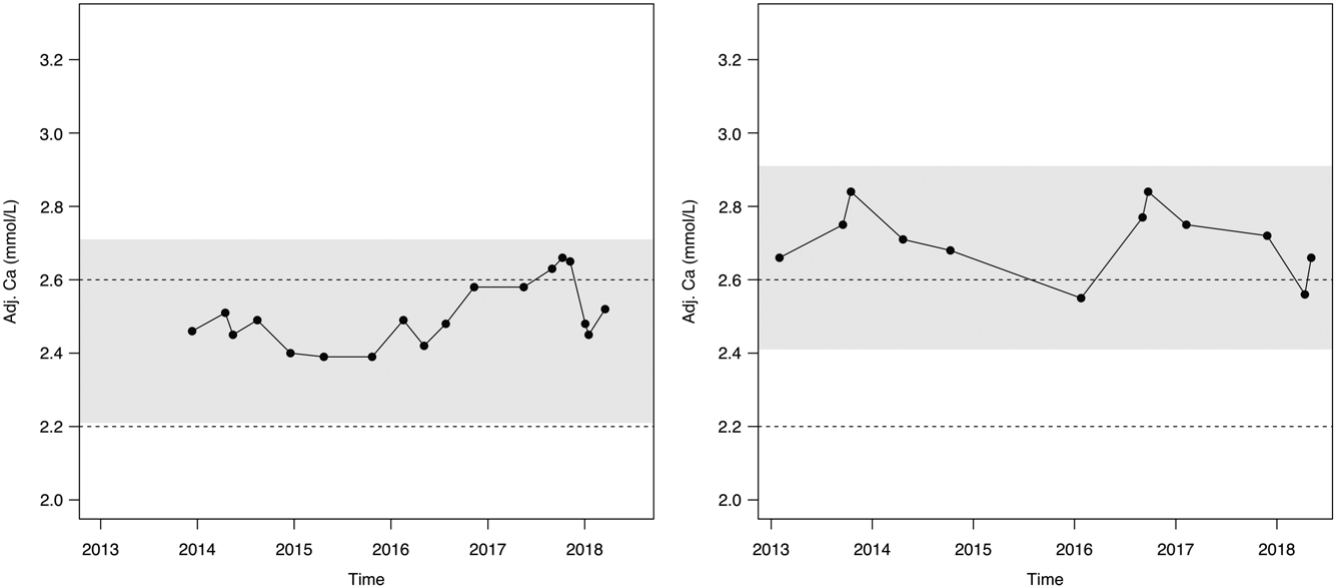
The follow up measurements from a patient with normocalcaemic hyperparathyroidism (NPHPT, left) and primary hyperparathyroidism (PHPT, right), both having intermittent hypercalcaemia yet all results falling with the LSC. The dashed lines represent the reference interval for calcium. The grey areas represent the least significant change (LSC: 0.25 and 0.24 mmol/L for NPHPT and PHPT, respectively); these are given around the first available measurement of calcium. Adj.Ca, adjusted calcium.

**Figure 2:**
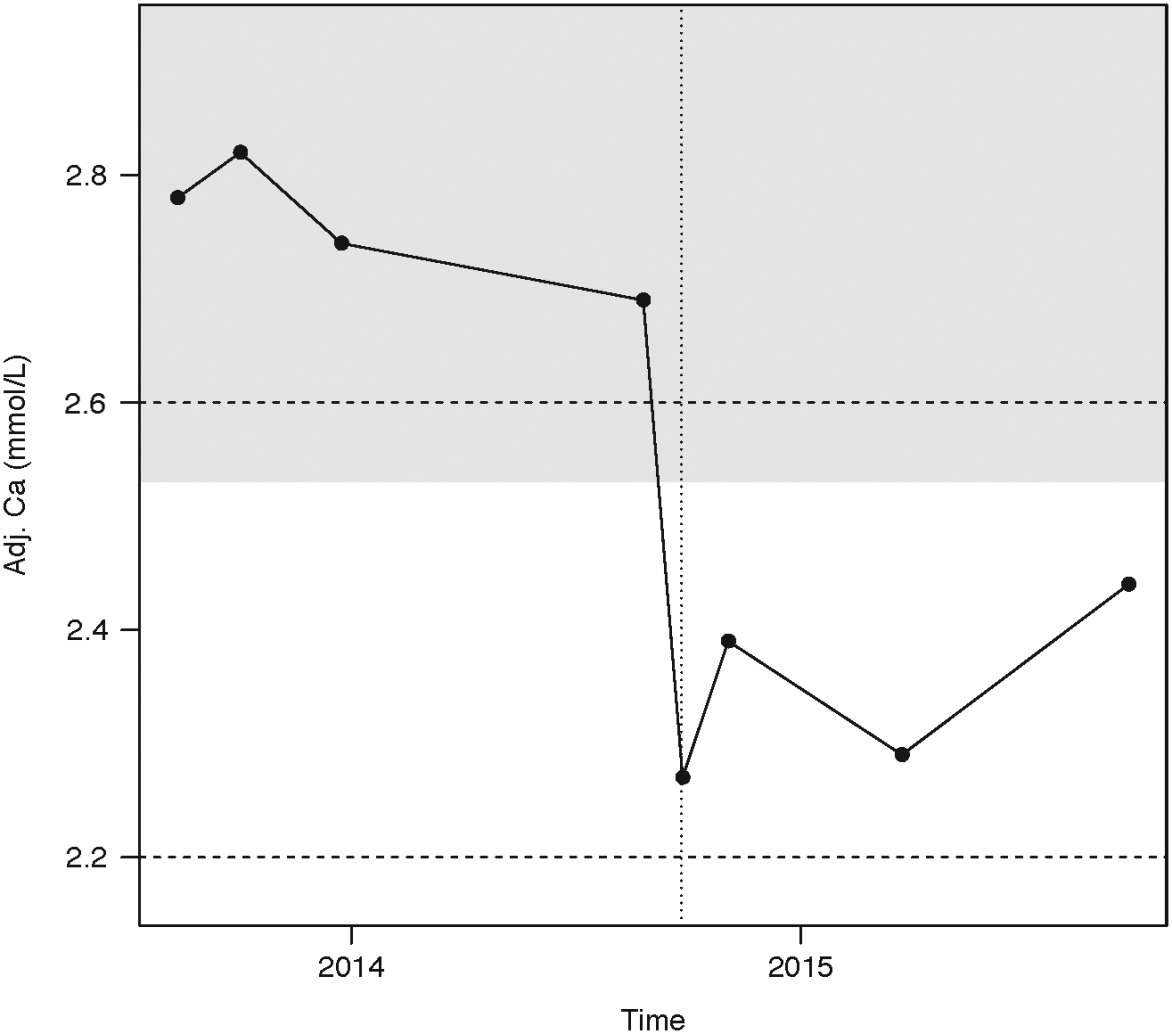
The follow up measurements from a patient with primary hyperparathyroidism patient (PHPT) who had parathyroid surgery (vertical dotted line). The dashed lines represent the reference interval for calcium. The grey area represents the least significant change (LSC: 0.24 mmol/L); this is given around the first available measurement of calcium. Adj.Ca, adjusted calcium. It can be seen that the postoperative calcium measurements all change by more than the LSC.

The least significant change calculated in this way could be used in two ways in these patients. First, it gives a range to which values are expected. For example, for a patient with NPHPT who has a baseline adjusted calcium of 2.55 mmol/L, the LSC is 0.25 mmol/L, so the next measurement would lie between 2.30 and 2.80 mmol/L, hence it is quite likely that a patient with apparent normocalcaemic hyperparathyroidism could have a subsequent measurement that is high, thus showing intermittent hypercalcaemia (11). Similarly, it is quite likely that a patient with apparent PHPT could have a subsequent measurement that is normal, again showing intermittent hypercalcaemia (Fig. 1). Moreover, it can be a marker of whether an individual has a significant change in adjusted calcium. Following surgery for PHPT, the physician can make sure that there was a statistically significant drop in calcium (Fig. 2).

We understand that the ideal way to establish the least significant change would require a more strict protocol. In our previous study, patients were not fasting and the intervals between calcium measurements were variable, so the within-subject standard deviation might not be as accurate. Moreover, the fact that we used a statistical approach to define our different groups might make the results specific to this population. As mentioned in that study, ideally, to give a more accurate estimate, a prospective observational study should be designed, having the same number of measurements for each patient at regular intervals (11). Another limitation is the lack of availability of ionised calcium measurements in our population. Despite these limitations, an approximate measure of the least significant change is provided for the first time in literature and can be used in clinical practice. We believe that more studies should be designed to provide a more accurate estimate in the future.

## Declaration of interest

The authors declare that there is no conflict of interest that could be perceived as prejudicing the impartiality of this brief report.

## Funding

M S received funding for her fellowship from the Medical Research Council Centre of Excellence for Musculoskeletal Ageing, from the Osteoporosis 2000 support group and from Roche Diagnostics. J S W Speaker's honoraria from Eli Lilly and Sandoz, grant funding from Alexion and Immunodiagnostic Systems, donation of drug from Eli Lilly, Prostrakan (Kyowa Kirin) and Consilient for clinical studies, donation of assay kits from Biomedica, consulting fees from Shire, Mereo Biopharma, Kyowa Kirin, UCB Pharma and PharmaCosmos. R E receives consultancy funding from IDS, Roche Diagnostics, GSK Nutrition, FNIH, Mereo, Lilly, Sandoz, Nittobo, Abbvie, Samsung, Haoma Medica and grant funding from Nittobo, IDS, Roche, Amgen and Alexion.
